# Clinical significance of tumor deposits in gastric cancer after radical gastrectomy: a propensity score matching study

**DOI:** 10.1186/s12957-023-03208-1

**Published:** 2023-10-13

**Authors:** Xiaohai Song, Kai Liu, Xuliang Liao, Yunfeng Zhu, BoQiang Peng, Weihan Zhang, Linyong Zhao, Xiaolong Chen, Kun Yang, Jiankun Hu

**Affiliations:** 1 Department of General Surgery and Laboratory of Gastric Cancer, State Key Laboratory of Biotherapy, West China Hospital, Sichuan University, No. 37 Guo Xue Xiang Street, Chengdu, 610041 Sichuan Province China; 2 Gastric Cancer Center, West China Hospital, Sichuan University, No. 37 Guo Xue Xiang Street, Chengdu, 610041 Sichuan Province China

**Keywords:** Gastric cancer, Prognosis, Tumor deposits, Propensity score matching, TNM stage

## Abstract

**Objective:**

The value of tumor deposits (TDs) in the prognosis and staging of gastric cancer (GC) is still under debate. This study aims to evaluate the prognostic value of TDs and the best ways to incorporate TDs in the TNM classification of GC.

**Methods:**

Patients (*n* = 3460) undergoing curative gastrectomy for GC in the West China Hospital from 2005 to 2017 were retrospectively reviewed and divided into two groups according to the TD status (positive vs. negative). Later, clinicopathological features and overall survival (OS) between the two groups were compared. Thereafter, the associations between the presence of TD and other clinicopathological factors were evaluated through logistic regression. In addition, univariate and multivariate Cox regression were conducted for determining prognostic factors. The possibility of selection bias was reduced through conducting the 1:1 propensity score matching (PSM) analysis. The modified classification systems proposed previously that incorporated TDs into the TNM staging system were assessed.

**Results:**

There were 10.5% of patients (362/3460) diagnosed with TDs. TDs were significantly related to unfavorable factors such as advanced T stage and N stage and independently associated with poor prognosis. The 5-year OS of patients with TDs was significantly lower than that of patients without TDs (31.0% vs. 60.9%, *P* < 0.001), whereas higher than that of patients with peritoneal metastasis (31.0% vs. 5.0%, *P* < 0.001). In patients receiving chemotherapy, the 5-year OS of patients with TDs was also significantly lower than that of patients without TDs (42.0% vs. 50.9%, *P* = 0.026). Moreover, the system incorporating TDs in the TNM classification as metastatic lymph nodes outperformed others.

**Conclusions:**

TDs are related to the aggressive characteristics and are an independent prognostic factor for GC. Incorporating TDs in the TNM classification as the metastatic lymph nodes increases the accuracy in predicting prognosis.

**Supplementary Information:**

The online version contains supplementary material available at 10.1186/s12957-023-03208-1.

## Introduction

Gastric cancer (GC) is a prevalent malignancy that ranks as the fifth most fatal cancer globally [1]. At present, the tumor node metastasis (TNM) classification system has been broadly applied in the prediction of prognosis and the determination of the optimal therapy alternatives of GC patients [2, 3]. In addition to the TNM classification system, many other clinicopathological parameters, such as tumor size, tumor deposits (TDs), or perineural invasion, have been additionally collected for prognosis prediction [3].

TDs, first recognized in colorectal cancer (CRC) in 1935 [4], are the satellite peritumoral nodules observed within lymph drainage area in the primary cancer with no identifiable lymph node tissue. Numerous subsequent studies have confirmed that TDs can be used to predict the prognosis of CRC [5–7]. Consequently, TDs are deemed to be N1c for CRC patients with no regional lymph node metastasis (LNM) in the TNM classification system (7th edition) [8]. In addition to CRC, TDs also exist in various other cancers, like GC, bile duct cancer, head and neck cancer, and pancreatic cancer [9, 10].

In recent years, with the gradual increase in the number of detected TDs in GC, increasing attention has been paid to the clinical significance of TDs. However, the value of TDs in the prognosis and staging of GC is still under debate. First, although previous studies have confirmed that TDs are an independent prognostic factor for GC, the clinical pathological characteristics related to TDs and the impact of TDs number on the prognosis remain controversial [11–18]. Additionally, TDs has been found to be closely related to the advanced TNM stage of GC; however, the prognostic significance of adjuvant chemotherapy in TD-positive GC is rarely reported. Third, whether TDs should be included in TNM classification and the way for its simple and reasonable incorporation in TNM classification remain unclear. At present, as suggested by the Japanese GC treatment guidelines, each TD can be considered one metastatic lymph node; therefore, it is incorporated in the N stage, but it is just an experience-based practice, and support from related clinical evidence is still lacking [19], while TDs are not incorporated into the TNM staging in the 8^th^ edition of the American Joint Committee on Cancer (AJCC) GC classification system. In addition, some researchers proposed to include TDs in the N stage [11, 14] or serosal invasion [13, 20], while others suggested that TDs with a number of > 2 or 3 should be categorized in the M1 stage [15, 21]. As a result, more investigations are needed to clarify the aforementioned aspects.

In the present retrospective work, 3460 GC patients undergoing radical surgery were examined for evaluating the correlations of TDs with clinicopathological characteristics and the prognosis of patients. Besides, the previously proposed methods incorporating TDs in the 8th TNM classification system were compared.

## Materials and methods

### Objects of study

**Fig. 1:**
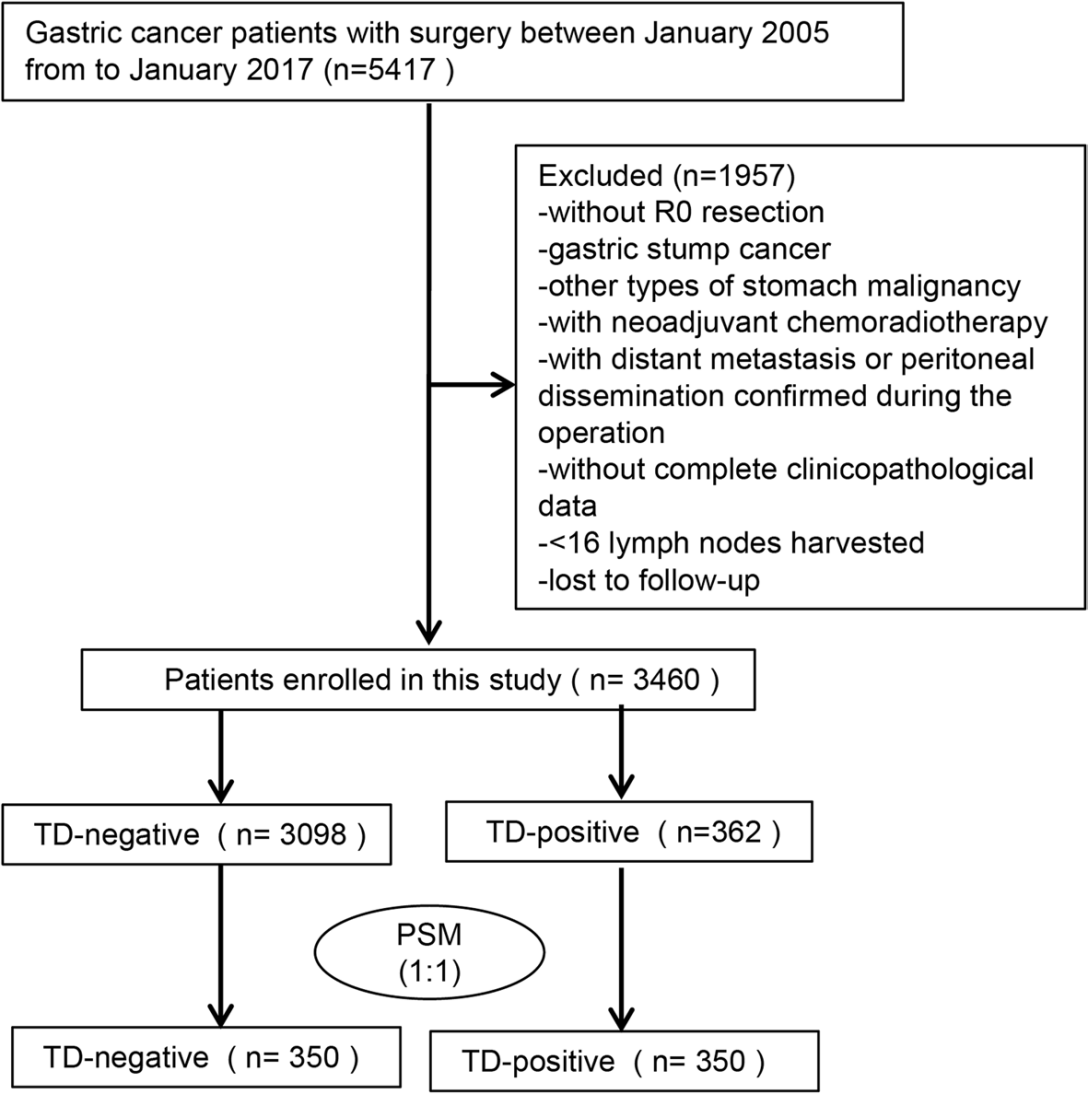
Flow chart of the study population. TD, tumor deposit; PSM, propensity score matching

From January 2005 to January 2017, 5417 GC patients receiving gastrectomy in the West China Hospital were reviewed in this study. Patients conforming to criteria below were included: (1) histologically confirmed adenocarcinoma and (2) radical gastrectomy (R0). Patients conforming to criteria below were eliminated: (1) those with other gastric malignancies, (2) gastric stump cancer, (3) those undergoing neoadjuvant chemoradiotherapy, (4) those with < 16 lymph nodes harvested, (5) without complete clinicopathological data, (6) lost to follow-up, and (7) with distant metastasis or peritoneal dissemination. Finally, 3460 cases were included into the present study. Figure 1 shows the flowchart of patient screening.

Among the 3460 patients, 1238 (35.7%) received adjuvant chemotherapy. In this study, 5-fluorouracil (FU) monotherapy or 5-FU and cisplatin combination therapy was the major adjuvant therapeutic regimen. Another 222 GC patients with peritoneal metastasis were included as a control group, and the prognosis was compared with that of the TD-positive group. Our study protocol gained approval from the ethics committee of the West China Hospital [2023 Review (842)]. Informed consents were obtained from all enrolled patients.

### TD definition

Pathologists from the Department of Pathology, West China Hospital were responsible for evaluating tumor histological sections. TD was defined in line with the AJCC GC classification system (8^th^ edition). Positive TDs were deemed to be discrete tumor cell foci discovered within lymphatic drainage area of primary cancers with no neural/vascular structures or lymph node tissues. TD number was also recorded. In addition, X-tile software (Version 3.6.1, Yale University) was used for calculating the best threshold TD number, which was determined to be 3, so as to investigate whether TD number could be used in prognosis prediction. Consequently, TD-positive patients were subsequently classified into two groups (1–3 and > 3 TDs).

### Follow-up

Each patient was followed up at 3-month intervals within the first year, at 6-month intervals from 2–5 years, and at 1-year intervals thereafter. During every follow-up, physical examination, abdominal ultrasound, computed tomography, and laboratory tests were completed. The overall survival (OS) was calculated from the date of surgery to the time of death from any cause or final follow-up (January 31, 2022). At the time of the last follow-up, 301 patients were lost, and they were excluded during the survival analysis.

### Statistical analysis

Clinicopathological factors in TD-positive group were compared with those in TD-negative group. Rank sum test and student’s *t*-test were adopted for comparing continuous variables, while chi-square test was applied in analyzing categorical variables. Univariate as well as multivariate logistic regression was performed for analyzing relations of TD status with other clinicopathological factors. OS was determined based on the Kaplan–Meier approach.

In addition, we also conducted one-to-one propensity score matching (PSM) analysis in both groups for reducing selection bias. Logistic regression was completed to estimate propensity scores, with variables of age, tumor location, histological type, Borrmann type, size, T stage, N stage, TNM stage, perineural and lymphovascular invasion, and chemotherapy being matching criteria and the caliper being 0.02.

Furthermore, the predictive abilities of different models were evaluated by the area under the ROC curve (AUC), area under the Harrell’s concordance index (C-index), and with Akaike information criterion (AIC), with the greater AUC and C-index data, whereas the lower AIC level indicating superior system discrimination ability. R statistical software package (version 4.2.1; R Project for Statistical Computing, Vienna, Austria) was employed for statistical analysis. *P* < 0.05 (two-sided) stood for statistical significance.

## Results

### Clinicopathological features

There were 2410 (69.7%) male individuals among the 3460 patients. They were classified into two groups based on TD status, with 362 (10.5%) in TD-positive group and 3098 (89.5%) in TD-negative group. There were 1018 TDs discovered among the 362 TD-positive patients and the number of TD ranged from 1 to 16. Among these patients, 144 cases had 1 TD, 86 cases had 2, 41 cases had 3, whereas 91 cases had more than 3 TDs.

The basic demographic and clinical characteristics of 3460 patients are shown in Table 1. TDs were found to be significantly associated with age, tumor location, tumor size, type of gastrectomy, Borrmann type, histologic type, advanced T stage, advanced N stage, advanced TNM stage, and perineural and lymphovascular invasion (all *P* < 0.05).

**Table 1:** Clinicopathological characteristics of gastric cancer patients with or without TD before and after PSM

**Factors**	**Before matching**			**After matching**		
	**TD (-) %**	**TD ( +) %**	***P***	**TD (-) %**	**TD ( +) %**	***P***
Gender			0.641			1.000
Male	2154 (69.5)	256 (70.7)		249 (71.1)	249 (71.1)	
Female	944 (30.5)	106 (29.3)		249 (71.1)	249 (71.1)	
Age			0.011			0.545
≤ 60 years	1647 (53.2)	167 (46.1)		172 (49.1)	164 (46.9)	
> 60 years	1451 (46.8)	195 (53.9)		178 (50.9)	186 (53.1)	
Tumor location			< 0.001			0.583
Upper	611 (19.7)	74 (20.4)		58 (16.6)	71 (20.3)	
Middle	335 (10.8)	38 (10.5)		39 (11.1)	37 (10.6)	
Lower	1697 (54.8)	164 (45.3)		162 (46.3)	161 (46.0)	
Two-thirds or more	455 (14.7)	86 (23.8)		91 (26.0)	81 (23.1)	
Type of gastrectomy			< 0.001			0.489
Distal	1927 (62.2)	174 (48.1)		185 (52.9)	172 (49.1)	
Proximal	383 (12.4)	33 (9.1)		139 (39.7)	145 (41.4)	
Total	788 (25.4)	155 (42.8)		26 (7.4)	33 (9.5)	
Tumor size			< 0.001			0.437
≤ 5 cm	1561 (50.4)	69 (19.1)		61 (17.4)	69 (19.7)	
> 5 cm	1537 (49.6)	293 (80.9)		289 (82.6)	281 (80.3)	
Borrmann type			< 0.001			0.760
I + II	1957 (63.2)	151 (41.7)		152 (43.4)	148 (42.3)	
III + IV	1141 (36.8)	211 (58.3)		198 (56.6)	202 (57.7)	
Histologic type			< 0.001			0.126
G1 + G2	813 (26.2)	40 (11.0)		28 (8.0)	40 (11.4)	
G3 + G4	2285 (73.8)	322 (89.0)		322 (92.0)	310 (88.6)	
T stage			< 0.001			0.178
T1	670 (21.6)	1 (0.3)		2 (0.6)	1 (0.3)	
T2	513 (16.6)	16 (4.4)		22 (6.3)	16 (4.6)	
T3	543 (17.5)	74 (20.4)		52 (14.9)	74 (21.1)	
T4a	1187 (38.3)	214 (59.1)		231 (66.0)	211 (60.3)	
T4b	185 (6.0)	57 (15.7)		43 (12.3)	48 (13.7)	
N stage			< 0.001			0.794
N0	1129 (36.4)	14 (3.9)		17 (4.9)	14 (4.0)	
N1	556 (17.9)	40 (11.0)		32 (9.1)	40 (11.4)	
N2	560 (18.1)	73 (20.2)		75 (21.4)	73 (20.9)	
N3a	586 (18.9)	146 (40.3)		140 (40.0)	145 (41.4)	
N3b	267 (8.6)	89 (24.6)		86 (24.6)	78 (22.3)	
pTNM stage			< 0.001			0.993
Stage I	818 (26.4)	4 (1.1)		3 (0.9)	4 (1.1)	
Stage II	797 (25.7)	23 (6.4)		25 (7.1)	23 (6.6)	
Stage IIIA	660 (21.3)	99 (27.3)		97 (27.7)	99 (28.3)	
Stage IIIB	498 (16.1)	123 (34.0)		124 (35.4)	123 (35.1)	
Stage IIIC	325 (10.5)	113 (31.2)		101 (28.9)	101 (28.9)	
Perineural invasion			< 0.001			0.069
Absence	2649 (85.5)	269 (74.3)		282 (80.6)	262 (74.9)	
Presence	449 (14.5)	93 (25.7)		68 (19.4)	88 (25.1)	
Lymphovascular invasion			< 0.001			0.225
Absence	2591 (83.6)	232 (64.1)		245 (70.0)	230 (65.7)	
Presence	507 (16.4)	130 (35.9)		105 (30.0)	120 (34.3)	
Chemotherapy			0.453			0.430
Absence	1996 (64.4)	226 (62.4)		230 (65.7)	220 (62.9)	
Presence	1102 (35.6)	136 (37.6)		120 (34.3)	130 (37.1)	

*Abbreviations*: *PSM* propensity score matching, *TD* tumor deposit

### Identification of risk factors for TD

By conducting logistic regression, the TD-related risk factors were identified. Through univariate regression, significant variables were identified to be age (*P* = 0.011), tumor size (*P* < 0.001), Borrmann type (*P* < 0.001), histological type (*P* < 0.001), T stage (*P* < 0.001), N stage (*P* < 0.001), perineural invasion (*P* < 0.001), and lymphovascular invasion (*P* < 0.001). Upon multivariate regression, age (*P* = 0.012), tumor size (*P* < 0.001), T stage (*P* < 0.001), N stage (*P* < 0.001), and lymphovascular invasion (*P* < 0.001) independently predicted the risk of TD occurrence (Table 2).

**Table 2:** Logistic regression analysis of the risk factors for the presence of TD

Variable	Univariate analysis			Multivariate analysis		
	**OR**	**95%CI**	***P***	**OR**	**95%CI**	***P***
Age	1.325	1.065–1.649	0.011	1.351	1.068–1.709	0.012
Tumor size	4.313	3.287–5.658	< 0.001	1.746	1.283–2.375	< 0.001
Borrmann type	2.397	1.921–2.990	< 0.001	-	-	-
Histologic type	2.864	2.042–4.017	< 0.001	-	-	-
T stage	2.183	1.931–2.468	< 0.001	1.504	1.296–1.746	< 0.001
N stage	2.002	1.827–2.194	< 0.001	1.578	1.418–1.755	< 0.001
Perineural invasion	2.040	1.579–2.635	< 0.001	-	-	-
Lymphovascular invasion	2.864	2.264–3.622	< 0.001	1.685	1.283–2.213	< 0.001

*Abbreviations*: *OR* odds ratio, *CI* confidence interval, *TD* tumor deposit

### Survival analysis

**Fig. 2:**
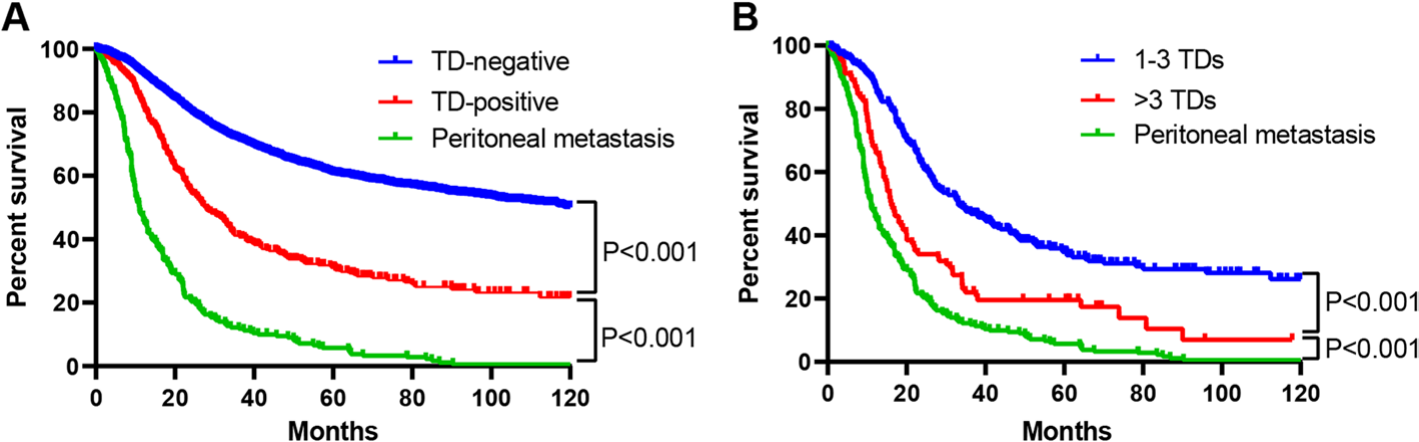
The Kaplan–Meier curves of overall survival (OS) of patients with positive TD, negative TD and peritoneal metastasis. **A** Comparison of survival curves between TD-negative, TD-positive patients, and those with peritoneal metastasis. **B** Comparison of survival curves among patients with different number of TDs and those with peritoneal metastasis. TD, tumor deposit

TD-positive patients had markedly decreased 3- and 5-year OS rates compared with TD-negative patients (40.2% vs. 71.6% and 31.0% vs. 60.9%, respectively; *P* < 0.001), whereas significantly superior survivals to patients developing peritoneal metastasis (40.2% vs. 11.0% and 31.0% vs. 5.0%, respectively; *P* < 0.001; Fig. 2A). To further elucidate the prognostic impact of TDs on GC patients, the 3- and 5-year OS rates were compared between patients with positive and negative TDs in each pN category and TNM stage. The results indicated that significant differences in survival were found between patients with and without TDs in N1 (*P* < 0.001), N3a (*P* < 0.001), and N3b (*P* = 0.005) category (Supplementary Fig. 1) and in stage IIIB (*P* < 0.001) and IIIC (*P* = 0.028) (Supplementary Fig. 2).

Additionally, survivals were significantly different among patients diagnosed with TDs to varying numbers. For patients who had 1–3 TDs, their 3- and 5-year OS rates significantly increased relative to those developing more than 3 TDs (47.3% vs. 22.0% and 34.8% vs. 19.5%, respectively; *P* < 0.001), while those in the latter were markedly superior to patients developing peritoneal metastasis (22.0% vs. 11.0% and 19.5% vs. 5.0%, respectively; *P* < 0.001; Fig. 2B). Upon multivariate regression, age (*P* < 0.001), tumor size (*P* = 0.003), type of gastrectomy (*P* = 0.013), T stage (*P* < 0.001), N stage (*P* < 0.001), TDs (*P* = 0.001), and chemotherapy (*P* < 0.001) were independently associated with the prognosis of all GC patients (Table 3 and Supplementary Table 1).

**Table 3:** Multivariate survival analysis of the patients following operation for gastric cancer before and after PSM

Variable	Before PSM			After PSM		
	**HR**	**95%CI**	***P***	**HR**	**95%CI**	***P***
Age (> 60 vs ≤ 60 years)	1.315	1.189–1.455	< 0.001	-	-	-
Tumor size (> 5 vs ≤ 5 cm)	1.202	1.063–1.361	0.003	1.338	1.016–1.761	0.038
Type of gastrectomy			0.013	-	-	-
Proximal vs distal	1.198	1.018–1.411	0.030	-	-	-
Total vs distal	1.398	1.115–1.753	0.004	-	-	-
T stage			< 0.001	-	-	
T2 vs T1	1.505	1.171–1.935	0.001	-	-	-
T3 vs T1	1.644	1.282–2.108	< 0.001	-	-	-
T4a vs T1	2.406	1.906–3.038	< 0.001	-	-	-
T4b vs T1	2.670	2.021–3.527	< 0.001	-	-	-
N stage			< 0.001	-	-	< 0.001
N1 vs N0	1.464	1.215–1.764	< 0.001	3.034	1.272–7.237	0.012
N2 vs N0	1.911	1.599–2.283	< 0.001	2.683	1.153–6.242	0.022
N3a vs N0	3.116	2.620–3.707	< 0.001	5.764	2.520–13.186	< 0.001
N3b vs N0	3.842	3.152–4.682	< 0.001	7.578	3.286–17.472	< 0.001
Chemotherapy (present vs absent)	0.667	0.598–0.745	< 0.001	0.759	0.621–0.929	0.007
TD (presence vs absence)	1.286	1.118–1.478	0.001	1.268	1.057–1.522	0.011

*Abbreviations*: *HR* hazard ratio, *CI* confidence interval, *TD* tumor deposit, *PSM* propensity score matching

For TD-positive patients, the prognosis of GC cases undergoing chemotherapy significantly improved relative to those not undergoing chemotherapy (*P* = 0.004) (Supplementary Fig. 3). Multivariate analysis indicated that N stage (*P* < 0.001) and TD number (*P* < 0.001) independently predicted the prognosis of patients receiving chemotherapy (Supplementary Table 2).

### PSM analysis

**Fig. 3:**
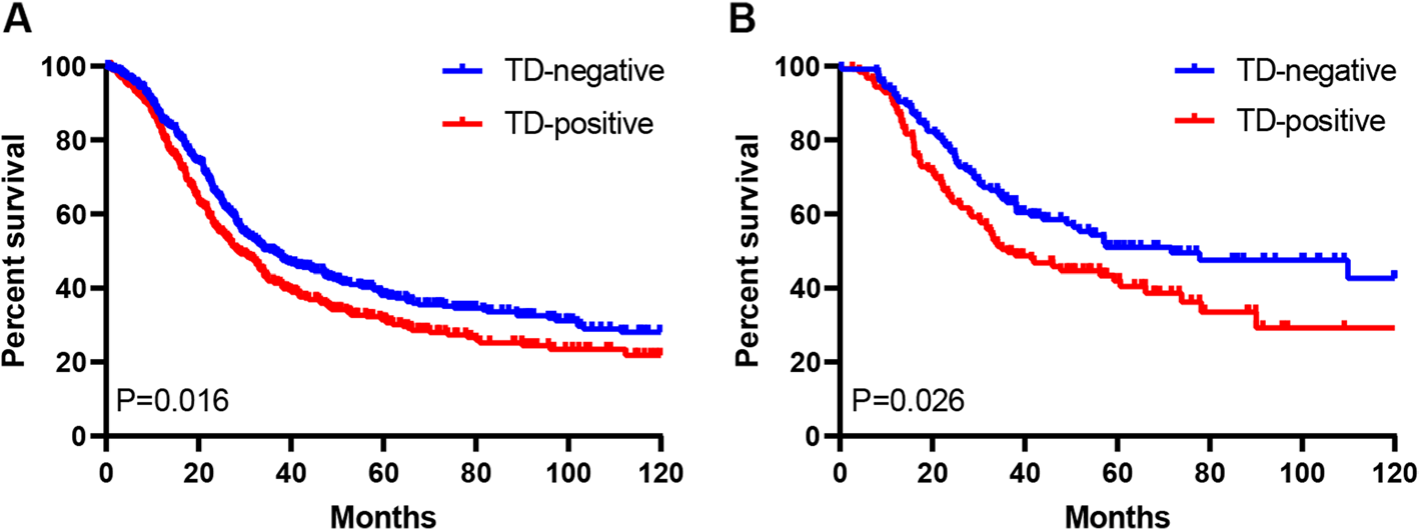
The Kaplan–Meier curves of overall survival (OS) of patients with positive and negative TD after PSM. **A** Comparison of survival curves between TD-negative and TD-positive patients after PSM. **B** Comparison of survival curves between TD-negative and TD-positive patients who received adjuvant chemotherapy after PSM. TD, tumor deposit; PSM, propensity score matching

In order to eliminate the impacts induced by confounding factors, the 1:1 PSM analysis was conducted for patients in both TD-positive and TD-negative groups (Supplementary Fig. 4). As a result, age, gender, tumor size, type of gastrectomy, tumor location, Borrmann type, histological type, T stage, N stage, TNM stage, perineural and lymphovascular invasion, and chemotherapy were not significantly different between two groups (Table 1). For TD-positive group, their 3- and 5-year OS rates remarkably decreased relative to TD-negative group (42.0% vs. 49.7% and 31.5% vs. 38.8%, respectively; *P* = 0.016; Fig. 3A). Upon multivariate regression, age (*P* = 0.002), T stage (*P* = 0.010), N stage (*P* < 0.001), chemotherapy (*P* < 0.001), and number of TDs (*P* < 0.001) independently predicted prognosis (Table 3 and Supplementary Table 1).

For patients who received adjuvant chemotherapy, the 1:1 PSM analysis was performed in 125 patients in both groups, according to age, gender, tumor location, type of gastrectomy, tumor size, Borrmann type, histological type, T stage, N stage, TNM stage, and perineural and lymphovascular invasion (Supplementary Table 3). In addition, TD-positive group showed significantly decreased 3- and 5-year OS rates compared with TD-negative group (49.5% vs. 63.9% and 42.0% vs. 50.9%, respectively; *P* = 0.026; Fig. 3B).

### Assessment of prediction accuracy for OS

**Fig. 4:**
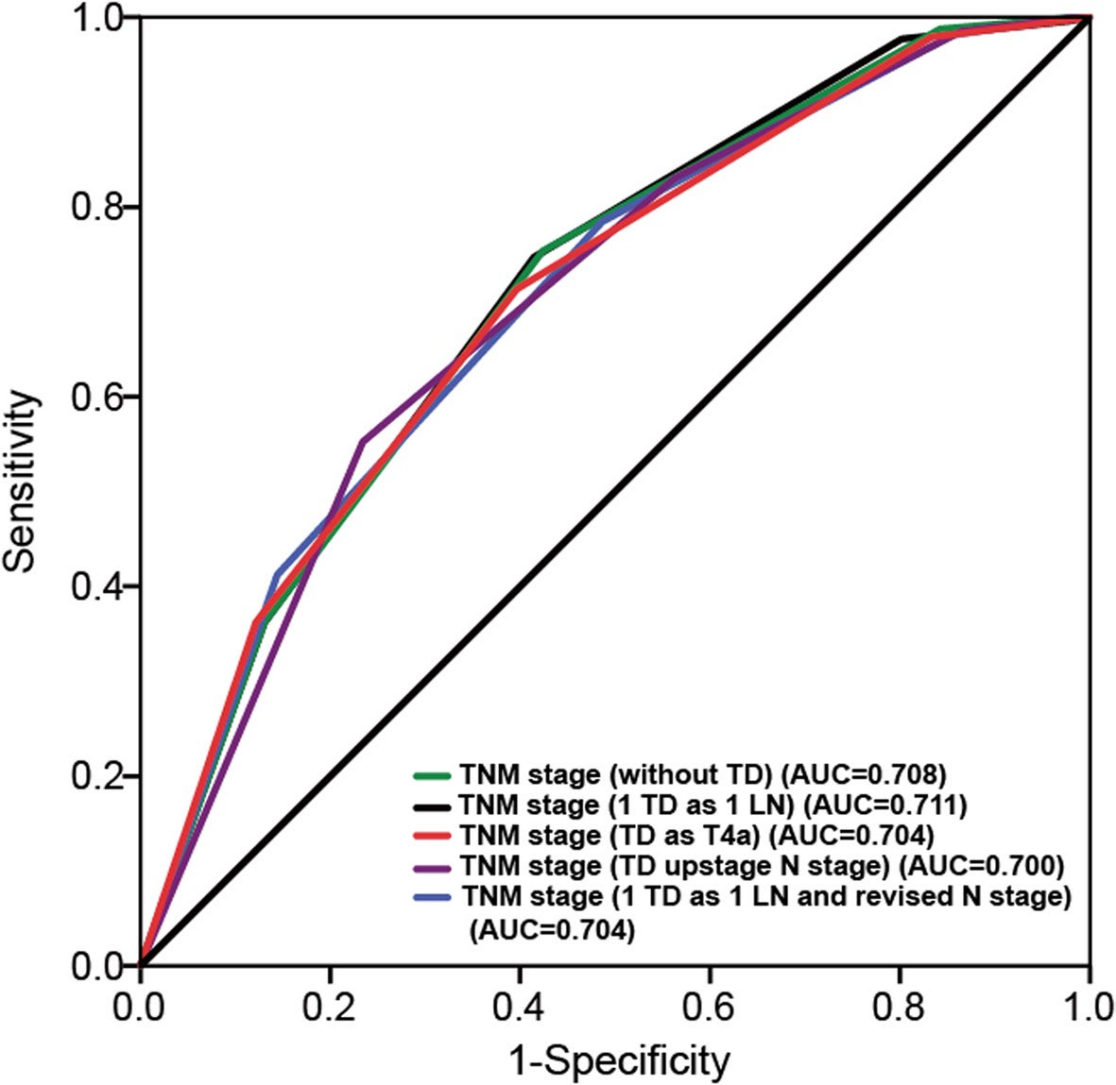
The area under receiver operating characteristic (ROC) curves (AUC) of different staging systems to predict the 5-year OS

This work compared the four different models with the 8th AJCC TNM classification system in terms of their prediction performance, with the greater AUC and C-index levels whereas the lower AIC level suggesting the superior discrimination. Typically, the classification system with TDs being metastatic lymph nodes exhibited the largest C-index and AUC values whereas the lowest AIC level (Table 4 and Fig. 4).

**Table 4:** Comparison of the performance between TNM staging system and other revised staging schemes after PSM

	**Description**	**AUC**	**95% CI**	**C-index**	**95% CI**	**AIC**
AJCC 8th	TNM staging system (Without TD)	0.708	0.666–0.750	0.641	0.616–0.666	818.87
AJCC 8th [19]	1 TD as 1 metastatic LN	0.711	0.669–0.752	0.643	0.619–0.667	817.65
Chen H. et al [11]	Presence of TDs upstage N stage except for N3b	0.700	0.658–0.743	0.639	0.615–0.663	826.80
Liang Y. et al [14]	1 TD as 1 positive LN; revised N category	0.704	0.662–0.745	0.640	0.615–0.665	824.73
Sun Z. et al [13]	Presence of TDs as T4a except for T4b	0.704	0.662–0.745	0.640	0.615–0.665	824.59

*Abbreviations: TD* tumor deposit, *CI* confidence interval, *AUC* area under the curve, *AIC* Akaike information criterion, *C-index* Harrell’s concordance index

## Discussion

In this study, 3460 GC patients undergoing radical gastrectomy were retrospectively analyzed for investigating the role of TDs. As a result, we found that TDs were associated with unfavorable clinicopathological factors and were an independent prognostic factor for GC patients. In addition, TDs were also related to poor survival of GC patients who received adjuvant chemotherapy. Besides, we found incorporating TDs in the TNM classification as the metastatic lymph nodes increased the accuracy in predicting prognosis of GC.

Among the patients enrolled in this study, the TD prevalence rate was 10.5%, similar to that in previous reports (10.5–27.5%) [12–14, 16, 20–23]. Furthermore, we found that TDs were related to the dismal clinicopathological factors that reflected disease aggressiveness. In the primary cohort before PSM analysis, TDs were significantly related to a number of variables (such as tumor location, tumor size, Borrmann type, histologic type, T stage, N stage, TNM stage, perineural and lymphovascular invasion), similar to results reported in previous studies [11, 12, 14]. Apart from the aforementioned clinicopathological features, TDs are also found related to distant metastasis [16]. As discovered by Etoh et al., TD-positive patients exhibited a higher propensity for presenting with peritoneal seeding at the time of surgery [24]. Therefore, TDs are an important indicator of cancer aggressiveness, and close follow-up is suggested for GC patients with TDs.

A mechanism underlying TD occurrence remains controversial in existing studies. In some studies, TDs were considered to be related to LNM extracapsular extensions or tumor overgrowth from the invading lymphovascular bundles, since TDs were reported to be significantly related to LNM [25–27]. However, other studies demonstrated that the primary lesion-derived tumor cells, which can spread to extramural or extranodal spaces directly, were the origin of TD [16, 24]. Logistic regression analysis of this study showed that TD occurrence was independently related to the larger tumor size, advanced lymph node stage, deeper tumor invasion, and lymphovascular invasion. Therefore, for non-serosal invading tumors, LNM and lymphovascular invasion may have an important effect on TD generation, while cell seeding release may lead to TD formation in serosal invading tumors. Interestingly, this study revealed a significant correlation between advanced age and TD occurrence. Analysis indicated that elderly gastric cancer patients tended to have deeper tumor invasion and larger tumor size (Supplementary Table 4), which might potentially contribute to TD occurrence.

As for the prognostic value of the TDs, previous studies have confirmed the positive influence of TDs on survival for GC [14, 17]. According to our results, TDs-positive GC patients also showed remarkably dismal survival compared with TD-negative patients, and similar findings were also observed in the sub-group analysis. In addition, TDs independently predicted patient prognosis upon multivariate regression both before and after PSM. However, whether TD number affects prognosis remains unclear. As discovered by Wang et al., GC patients diagnosed with 1 and 2 TDs shared similar survival, but the survival markedly decreased in patients with ≥ 3 TDs after curative operation [15], as validated by another study in 2022 [28]. Similarly, Sun et al. [13] and Etoh et al. [24] came to consistent findings, but they used diverse thresholds (1, 2–3, > 3 vs. 0, 1–4, ≥ 5, respectively). Unlike these studies, both Anup et al. [20] and Zhou et al. [12] suggested that the TD number was not related to prognosis. In our study, GC patients having > 3 TDs exhibited poorer survival than those with 1–3 TDs. The reasons for these discrepancies may be the different tumor stages among GC patients and the inconsistent criteria for grouping based on TD numbers. Consequently, TD number should not be neglected in the clinical prognosis evaluation and the determination of therapeutic strategy for GC patients. Wang et al. indicated that GC patient who have ≥ 3 TDs shared similar survival to stage IV patients and should be treated as M1 stage [15], while Sun et al. found that patients having > 3 TDs had superior survival to those developing peritoneal metastases [13]. Our result was consistent with the results obtained by Sun et al., as patients having > 3 TDs had superior prognosis to patients with peritoneal metastasis, which demonstrated that TD-positive GC should be treated as a locally advanced, rather than stage IV disease.

Even though TDs occurrence is related to adverse clinicopathological factors and poor prognosis of GC patients, there remains controversy regarding whether adjuvant chemotherapy can improve the prognosis of patients with TDs. As reported by one retrospective study conducted in China, the 5-year survival of T1–T2 stage TD-positive patients undergoing chemotherapy remarkably increased compared with patients who did not receive chemotherapy [29]. However, Xu et al. demonstrated that survival was not significantly different in chemotherapy compared with non-chemotherapy groups among the 11 T1–T2 stage TD-positive GC patients, which was possibly associated with the small sample size [28]. Based on our study, TD-positive cases who underwent radical gastrectomy plus adjuvant chemotherapy achieved the better survival, suggesting that the effectiveness of complete removal of adipose connective tissue through D2/R0 surgery and adjuvant chemotherapy is highly recommended for patients with TDs. Furthermore, some researchers have studied whether TDs could affect the prognosis of GC patients receiving adjuvant chemotherapy. For example, Xu et al. reported that for patients with T1–T2 stage who received chemotherapy, the TD-positive patients had markedly decreased 5-year survival rate compared with TD-negative GC patients [28]. In addition, Kim et al. also found that TDs predicted dismal prognosis of GC patients receiving adjuvant chemotherapy [16]. Different from their study, in this paper, PSM analysis was used for examining how TDs affected survival of GC patients who received chemotherapy, which could eliminate the impacts induced by confounding factors. Our results indicated that even after PSM, TD-positive GC patients still exhibited the poor survival compared with TD-negative patients who were treated with adjuvant chemotherapy. Therefore, more individualized treatments should be administered to TD-positive GC patients in the future.

To date, it is still unknown about the best way to incorporate TDs in the AJCC TNM classification system for GC. As suggested by the Japanese GC treatment guidelines, TDs should be considered as metastatic lymph nodes in determining pN, but sufficient clinical evidence is lacking [19]. Some studies have also investigated additional practicable approaches for efficiently incorporating TDs in the TNM classification system. According to Wang et al., TD should be incorporated in N3 or M1 stage according to the TD number collected [15]. However, according to the results of our study and those of Sun et al., TDs-positive patients showed the superior prognostic outcome to those developing peritoneal metastasis, indicating that TDs should not be considered as peritoneal metastases, regardless of their number. In Sun et al.’s study, TD was deemed as the serosal invasion form (T4a), since TD-positive patients of pT1-4a category did not have significantly different prognosis compared with TD-negative patients of pT4a category [13], as verified in another article conducted in 2017 [20]. On the contrary, Lee et al. suggested that TDs must be incorporated into the N category and considered positive lymph nodes [16]. The authors also put forward the revised N stage standard where total metastasis number was determined through the addition of metastatic lymph nodes and perigastric TDs numbers. In addition, as put forward by Chen et al., TDs occurrence must upstage N category except for N3b [11]. Furthermore, Gu et al. put forward the revised scheme in TD-positive patients through including TDs in TNM classification system, where the current classification must be upstaged with the exception of stage IIIC patients [17]. The present work assessed the previous proposals of incorporating TDs into T or N categories based on our data; as a result, the system that counted TDs as metastatic lymph nodes had improved prediction accuracy, consistent with the Japanese GC treatment guidelines. The findings of this study remind both pathologists and clinicians to pay more attention to TDs of GC. Incorporating TDs into the TNM staging system can enhance the accuracy of gastric cancer prognosis prediction, thereby assisting clinicians in formulating appropriate treatment strategies. However, including TDs in TNM classification system requires more research. Most of the present proposals are based on single-center databases, with small sample size and without any external validation. In addition, the TD detection rate is quite low during the early T and N categories, making it difficult to evaluate the value of TDs. Consequently, more large-scale multicenter studies are warranted for verifying the above models and developing a more appropriate proposal for incorporating TDs in the TNM classification system.

This work focused on examining whether TDs could be used in prognosis prediction, rather than providing the new classification strategy incorporating TDs for GC. Certain limitations must be noted in this work. Firstly, this was a retrospective study, there might be some selection bias of patients. To minimize the effects of selection bias, on one hand, we have made utmost efforts to conduct follow-ups, minimizing the rate of loss to follow-up. In this study, the loss to follow-up rate was 5.6%. On the other hand, PSM analysis was conducted to reduce the impacts of potential confounders and selection bias. Secondly, some variables, such as Lauren classification, anatomic location of TDs, patterns of TDs, and recurrence rate were unavailable in some patients; therefore, these variables were not incorporated for analysis. Thirdly, because our work only focused on locally advanced GC cases, the relationship between TDs and M1 stage was not analyzed. At last, all patients in this study were enrolled from a single institution, and our results should be further confirmed in large-scale studies, especially the perspective ones.

## Conclusions

To sum up, TDs occurrence is related to the aggressive characteristics of GC. Further, TDs independently predict the lower OS in GC cases. Incorporating TDs in the TNM classification system as metastatic lymph nodes helps to increase the accuracy in prognosis prediction; nonetheless, it is still necessary to explore the suitable incorporation approach and verify it with the large-scale, prospective cohort studies. Considering their clinical importance, TDs should be collected and analyzed to thoroughly assess their value in GC in the future.

### Supplementary Information

**Additional file 1.**

**Additional file 2.**

**Additional file 3.**

**Additional file 4.**

**Additional file 5.**

**Additional file 6.**

**Additional file 7.**

**Additional file 8.**

## Data Availability

Data analyzed in this study are available from the corresponding author upon reasonable request.

## Abbreviations

GC: Gastric cancer
TDs: Tumor deposits
PSM: Propensity score matching
CRC: Colorectal cancer
OS: Over survival
AJCC: The American Joint Committee on Cancer
TNM: Tumor node metastasis
LNM: Lymph node metastasis
AUC: Area under the ROC curve
AIC: Akaike information criterion
