# Supplementary figures and images for: Clinical significance of tumor deposits in gastric cancer after radical gastrectomy: a propensity score matching study
Source: World J Surg Oncol. 2023 Oct 13;21:325. doi: 10.1186/s12957-023-03208-1 (PMC10571457; doi:10.1186/s12957-023-03208-1)

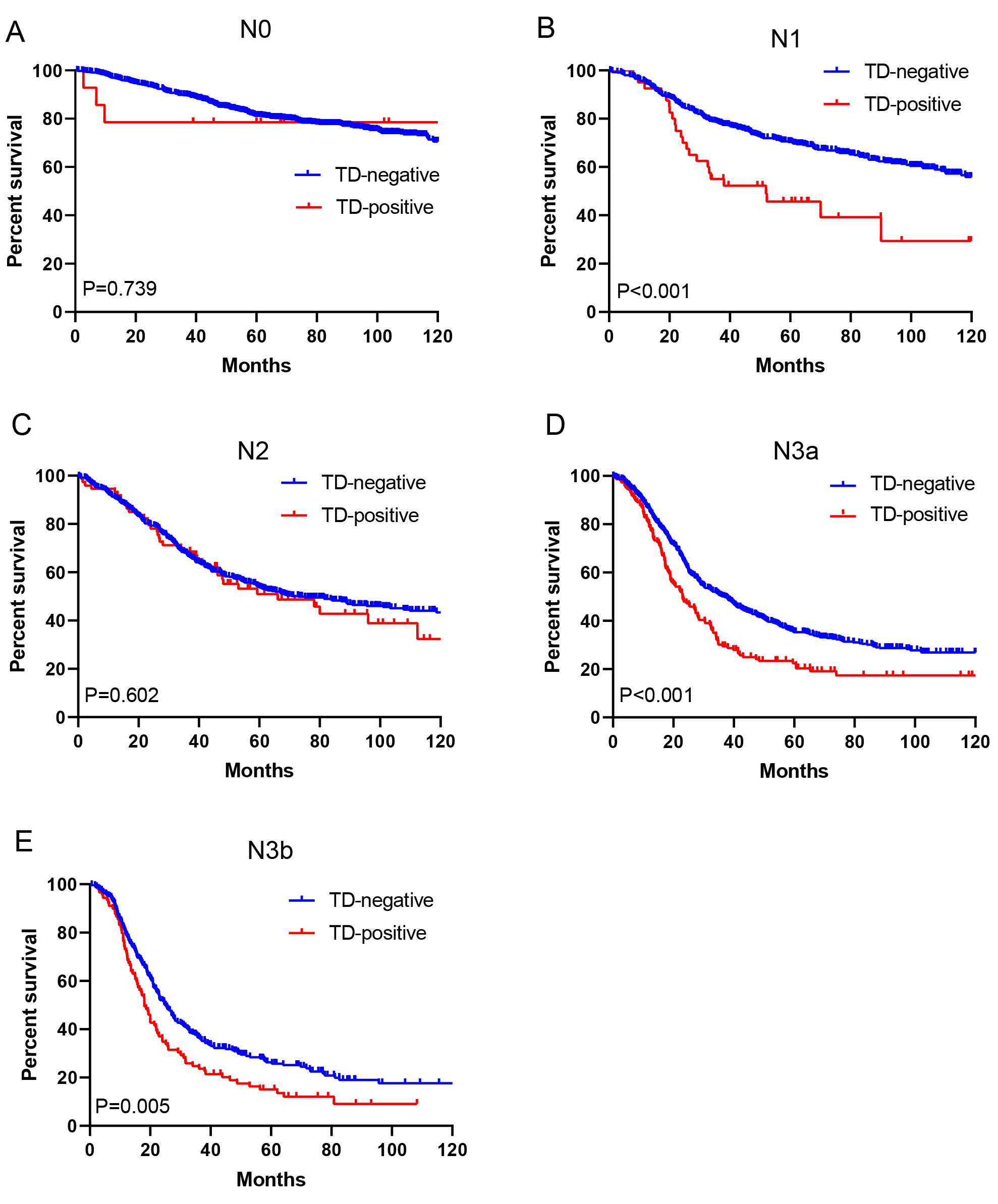

Supplement: Supplementary file 1 — Additional file 1. [file 12957_2023_3208_MOESM1_ESM.png]

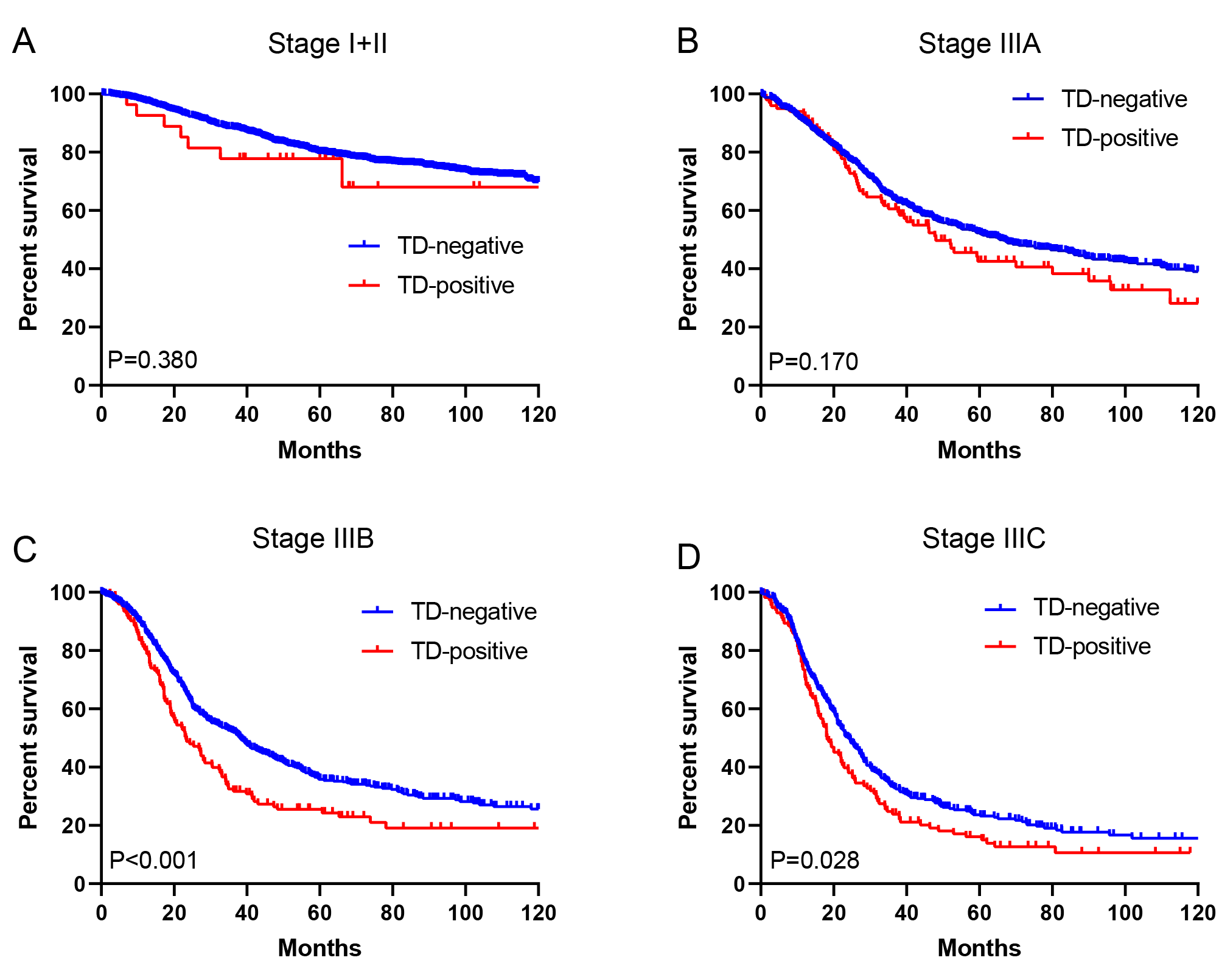

Supplement: Supplementary file 2 — Additional file 2. [file 12957_2023_3208_MOESM2_ESM.png]

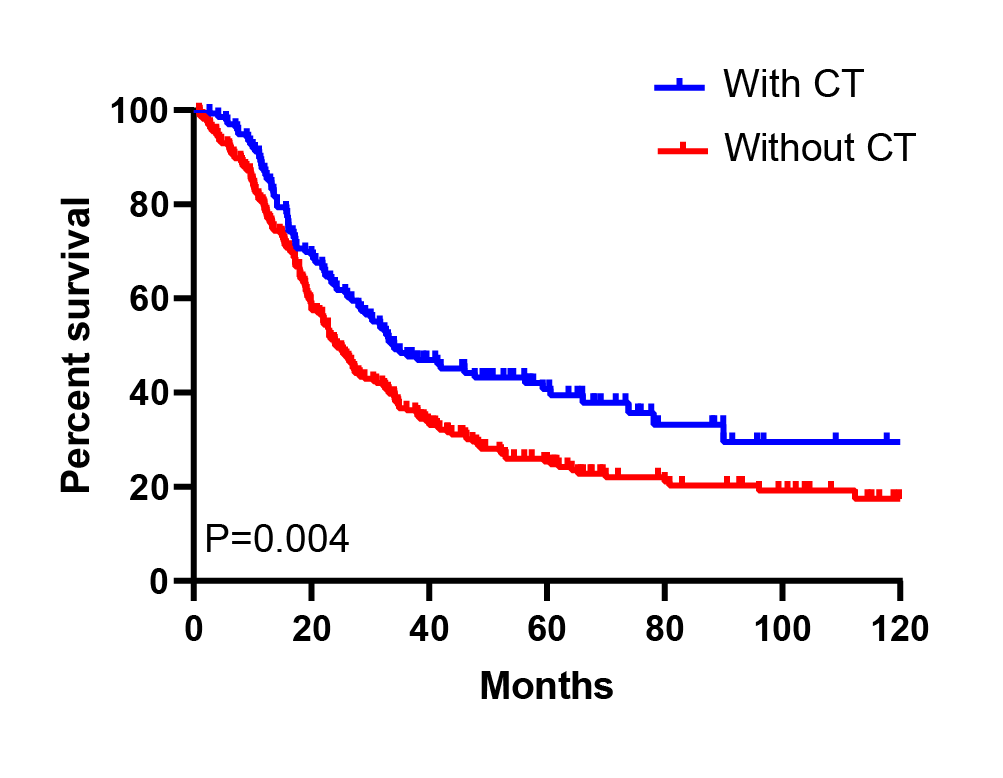

Supplement: Supplementary file 3 — Additional file 3. [file 12957_2023_3208_MOESM3_ESM.png]

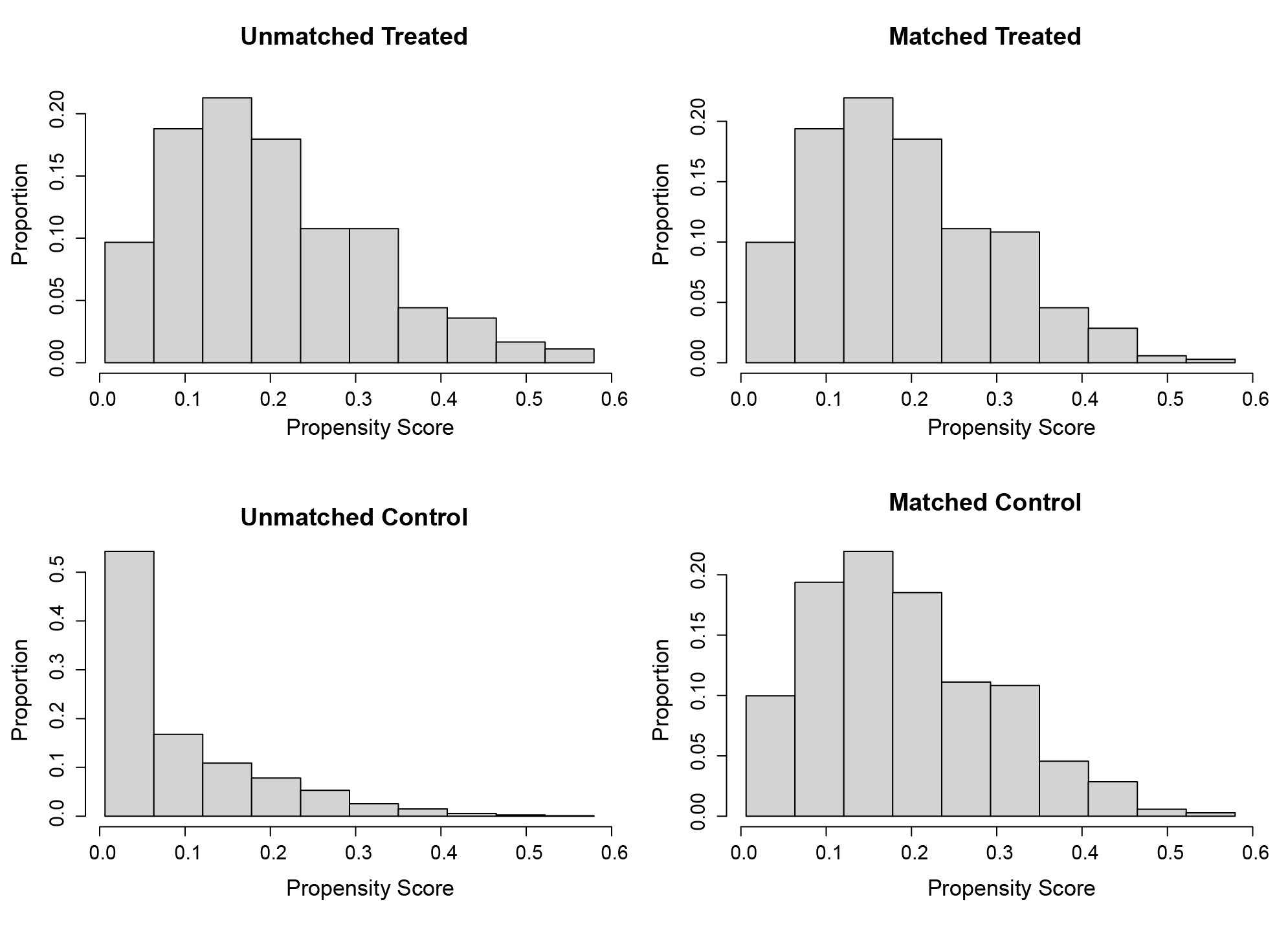

Supplement: Supplementary file 4 — Additional file 4. [file 12957_2023_3208_MOESM4_ESM.png]
