# Supplementary material for: Clinical significance of tumor deposits in gastric cancer after radical gastrectomy: a propensity score matching study
Source: World J Surg Oncol. 2023 Oct 13;21:325. doi: 10.1186/s12957-023-03208-1 (PMC10571457; doi:10.1186/s12957-023-03208-1)
Supplement: Supplementary file 5 — Additional file 5. [file 12957_2023_3208_MOESM5_ESM.docx]

| **Supplementary table 1** Univariate survival analysis of the patients following operation for gastric cancer before and after PSM | | | | | | |
| --- | --- | --- | --- | --- | --- | --- |
| **Variable** | **Before PSM** | | | **After PSM** | | |
|  | **HR** | **95%CI** | ***P*** | **HR** | **95%CI** | ***P*** |
| Age (>60 vs ≤60 yrs) | 1.301 | 1.179-1.436 | < 0.001 |  |  |  |
| Gender (Female vs Male) | 0.917 | 0.823-1.022 | 0.119 |  |  |  |
| Location |  |  | < 0.001 |  |  |  |
| Middele vs Upper | 0.907 | 0.755-1.090 | 0.298 |  |  |  |
| Lower vs Upper | 0.748 | 0.658-0.851 | < 0.001 |  |  |  |
| Two-thirds or more vs Upper | 1.439 | 1.238-1.673 | < 0.001 |  |  |  |
| Type of gastrectomy |  |  | < 0.001 |  |  |  |
| Proximal vs Distal | 1.828 | 1.640-2.038 | < 0.001 |  |  |  |
| Total vs Distal | 1.426 | 1.231-1.653 | < 0.001 |  |  |  |
| Tumor size (>5 vs ≤5cm) | 2.471 | 2.223-2.747 | < 0.001 | 1.373 | 1.048-1.800 | 0.022 |
| Borrmann type (III+IV vs I+II) | 1.962 | 1.778-2.166 | < 0.001 |  |  |  |
| Histologic type (G3+G4 vs G1+G2 ) | 1.985 | 1.735-2.270 | < 0.001 |  |  |  |
| T stage |  |  | < 0.001 | - | - | - |
| T2 vs T1 | 2.106 | 1.655-2.680 | 0.001 | - | - | - |
| T3 vs T1 | 2.913 | 2.322-3.654 | < 0.001 | - | - | - |
| T4a vs T1 | 5.506 | 4.520-6.708 | < 0.001 | - | - | - |
| T4b vs T1 | 8.427 | 6.658-10.665 | < 0.001 | - | - | - |
| N stage |  |  | < 0.001 | - | - | < 0.001 |
| N1 vs N0 | 1.868 | 1.561-2.236 | < 0.001 | 2.969 | 1.247-7.068 | 0.014 |
| N2 vs N0 | 2.836 | 2.404-3.345 | < 0.001 | 2.625 | 1.131-6.088 | 0.025 |
| N3a vs N0 | 5.140 | 4.417-5.981 | < 0.001 | 5.758 | 2.525-13.130 | < 0.001 |
| N3b vs N0 | 7.267 | 6.124-8.625 | < 0.001 | 7.580 | 3.300-17.414 | < 0.001 |
| Perineural invasion (Presence vs absence) | 1.402 | 1.225-1.604 | < 0.001 |  |  |  |
| Lymphovascular invasion (Presence vs absence) | 1.602 | 1.423-1.803 | < 0.001 |  |  |  |
| Chemotherapy (Presence vs absence) | 0.684 | 0.613-0.762 | < 0.001 | 0.769 | 0.630-0.940 | 0.010 |
| TD (Presence vs absence) | 2.449 | 2.141-2.800 | < 0.001 | 1.276 | 1.063-1.531 | 0.009 |
| *Abbreviations*: *HR* hazard ratio, *CI* confidence interval, *TD* tumor deposit, *PSM* propensity score matching. | | | | | | |
