# Supplementary material for: Clinical significance of tumor deposits in gastric cancer after radical gastrectomy: a propensity score matching study
Source: World J Surg Oncol. 2023 Oct 13;21:325. doi: 10.1186/s12957-023-03208-1 (PMC10571457; doi:10.1186/s12957-023-03208-1)
Supplement: Supplementary file 6 — Additional file 6. [file 12957_2023_3208_MOESM6_ESM.docx]

| **Supplementary table2** Univariate and multivariate survival analysis of gastric cancer patients with TD | | | | | | |
| --- | --- | --- | --- | --- | --- | --- |
| **Variable** | **Univariate analysis** | | | **Multivariate analysis** | | |
|  | **HR** | **95%CI** | ***P*** | **HR** | **95%CI** | ***P*** |
| Location |  |  | 0.010 |  |  |  |
| Upper vs lower | 1.788 | 1.115-2.868 | 0.016 |  |  |  |
| Middle vs lower | 1.539 | 1.083-2.186 | 0.016 |  |  |  |
| Two-thirds or more vs lower | 1.882 | 1.286-2.755 | 0.001 |  |  |  |
| Tumor size (>5 vs ≤5cm) | 1.570 | 1.117-2.209 | 0.009 |  |  |  |
| T stage |  |  | 0.018 |  |  |  |
| T3 vs T1+2 | 1.219 | 0.594-2.502 | 0.589 |  |  |  |
| T4a vs T1+2 | 1.694 | 0.865-3.315 | 0.124 |  |  |  |
| T4b vs T1+2 | 2.189 | 1.027-4.468 | 0.031 |  |  |  |
| N stage |  |  | < 0.001 |  |  | < 0.001 |
| N1 vs N0 | 3.003 | 0.901-10.008 | 0.073 | 2.935 | 0.877-9.826 | 0.081 |
| N2 vs N0 | 2.523 | 0.779-8.176 | 0.123 | 2.492 | 0.767-8.093 | 0.129 |
| N3a vs N0 | 5.921 | 1.879-18.651 | 0.002 | 5.134 | 1.619-16.286 | 0.005 |
| N3b vs N0 | 7.798 | 2.457-24.753 | < 0.001 | 6.170 | 1.922-19.801 | 0.002 |
| Chemotherapy (Present vs absent) | 0.686 | 0.528-0.891 | 0.005 |  |  |  |
| TD number (>3 vs ≤3) | 1.992 | 1.522-2.606 | < 0.001 | 1.738 | 1.313-2.301 | < 0.001 |
| *Abbreviations*: *HR* hazard ratio, *CI* confidence interval, *TD* tumor deposit. | | | | | | |
