# Supplementary material for: Clinical significance of tumor deposits in gastric cancer after radical gastrectomy: a propensity score matching study
Source: World J Surg Oncol. 2023 Oct 13;21:325. doi: 10.1186/s12957-023-03208-1 (PMC10571457; doi:10.1186/s12957-023-03208-1)
Supplement: Supplementary file 7 — Additional file 7. [file 12957_2023_3208_MOESM7_ESM.docx]

| **Supplementary table 3** Clinicopathological characteristics of gastric cancer patients receivig chemotherapy with or without TD before and after PSM | | | | | | |
| --- | --- | --- | --- | --- | --- | --- |
|  | **Before matching** | | | **After matching** | | |
| **Factors** | **TD (-) %** | **TD (+) %** | ***P*** | **TD (-) %** | **TD (+) %** | ***P*** |
| Gender |  |  | 0.355 |  |  | 0.582 |
| Male | 764 (69.3) | 89 (65.4) |  | 89 (71.2) | 85 (68.0) |  |
| Female | 338 (30.7) | 47 (34.6) |  | 36 (28.8) | 40 (32.0) |  |
| Age |  |  | 0.044 |  |  | 0.613 |
| ≤60 yrs | 619 (56.2) | 64 (47.1) |  | 66 (52.8) | 62 (49.6) |  |
| >60 yrs | 483 (43.8) | 72 (52.9) |  | 59 (47.2) | 63 (50.4) |  |
| Tumor Location |  |  | 0.002 |  |  | 0.607 |
| Upper | 220 (20.0) | 37 (27.2) |  | 29 (23.2) | 34 (27.2) |  |
| Middle | 118 (10.7) | 12 (8.8) |  | 16 (12.8) | 10 (8.0) |  |
| Lower | 619 (56.2) | 57 (41.9) |  | 53 (42.4) | 55 (44.0) |  |
| Two-thirds or more | 145 (13.1) | 30 (22.1) |  | 27 (21.6) | 26 (20.8) |  |
| Type of gastrectomy |  |  | < 0.001 |  |  | 0.538 |
| Distal | 697 (63.3) | 60 (44.1) |  | 59 (47.2) | 58 (46.4) |  |
| Proximal | 298 (27.0) | 62 (45.6) |  | 57 (45.6) | 53 (42.4) |  |
| Total | 107 (9.7) | 14 (10.3) |  | 9 (7.2) | 14 (11.2) |  |
| Tumor size |  |  | < 0.001 |  |  | 0.880 |
| ≤5cm | 539 (48.9) | 29 (21.3) |  | 28 (22.4) | 29 (23.2) |  |
| >5cm | 563 (51.1) | 107 (78.7) |  | 97 (77.6) | 96 (76.8) |  |
| Borrmann type |  |  | < 0.001 |  |  | 0.696 |
| I+II | 659 (59.8) | 50 (36.8) |  | 46 (36.8) | 49 (39.2) |  |
| III+IV | 443 (40.2) | 86 (63.2) |  | 79 (63.2) | 76 (60.8) |  |
| Histologic type |  |  | 0.003 |  |  | 0.859 |
| G1+G2 | 280 (25.4) | 19 (14.0) |  | 19 (15.2) | 18 (14.4) |  |
| G3+G4 | 822 (74.6) | 117 (86.0) |  | 106 (84.8) | 107 (85.6) |  |
| T stage |  |  | < 0.001 |  |  | 0.457 |
| T1 | 166 (15.1) | 1 (0.7) |  | 3 (2.4) | 1 (0.8) |  |
| T2 | 217 (19.7) | 8 (5.9) |  | 7 (5.6) | 8 (6.4) |  |
| T3 | 255 (23.1) | 37 (27.2) |  | 27 (21.6) | 37 (29.6) |  |
| T4a | 421 (38.2) | 71 (52.2) |  | 77 (61.6) | 66 (52.8) |  |
| T4b | 43 (3.9) | 19 (14.0) |  | 11 (8.8) | 13 (10.4) |  |
| N stage |  |  | < 0.001 |  |  | 0.966 |
| N0 | 346 (31.4) | 6 (4.4) |  | 7 (5.6) | 6 (4.8) |  |
| N1 | 241 (21.9) | 19 (14.0) |  | 17 (13.6) | 19 (15.2) |  |
| N2 | 221 (20.1) | 31 (22.8) |  | 28 (22.4) | 31 (24.8) |  |
| N3a | 217 (19.7) | 53 (39.0) |  | 51 (40.8) | 50 (40.0) |  |
| N3b | 77 (7.0) | 27 (19.9) |  | 22 (17.6) | 19 (15.2) |  |
| pTNM stage |  |  | < 0.001 |  |  | 0.941 |
| Stage I | 209 (19.0) | 2 (1.5) |  | 1 (0.8) | 2 (1.6) |  |
| Stage II | 383 (34.8) | 14 (10.3) |  | 16 (12.8) | 14 (11.2) |  |
| Stage IIIA | 240 (21.8) | 36 (26.5) |  | 33 (26.4) | 36 (28.8) |  |
| Stage IIIB | 181 (16.4) | 50 (36.8) |  | 48 (38.4) | 49 (39.2) |  |
| Stage IIIC | 89 (8.1) | 34 (25.0) |  | 27 (21.6) | 24 (19.2) |  |
| Perineural invasion |  |  | 0.002 |  |  | 0.576 |
| Absence | 883 (80.1) | 93 (68.4) |  | 91 (72.8) | 87 (69.6) |  |
| presence | 219 (19.9) | 43 (31.6) |  | 34 (27.2) | 38 (30.4) |  |
| Lymphovascular invasion |  |  | < 0.001 |  |  | 0.783 |
| Absence | 917 (83.2) | 90 (66.2) |  | 37 (29.6) | 39 (31.2) |  |
| presence | 185 (16.8) | 46 (33.8) |  | 88 (70.4) | 86 (68.8) |  |
| *Abbreviations: PSM* propensity score matching, *TD* tumor deposit. | | | | | | |
