# Supplementary material for: Clinical significance of tumor deposits in gastric cancer after radical gastrectomy: a propensity score matching study
Source: World J Surg Oncol. 2023 Oct 13;21:325. doi: 10.1186/s12957-023-03208-1 (PMC10571457; doi:10.1186/s12957-023-03208-1)
Supplement: Supplementary file 8 — Additional file 8. [file 12957_2023_3208_MOESM8_ESM.docx]

| **Supplementary table 4** Relationship between age and clinicopathological characteristics of patients with gastric cancer. | | | |
| --- | --- | --- | --- |
| **Factors** | ≤60 yrs **n (%)** | >60 yrs **n (%)** | ***P*** |
| Gender |  |  |  |
| Male | 1169 (64.4) | 1241 (75.4) | < 0.001 |
| Female | 645 (35.6) | 405 (24.6) |  |
| Tumor Location |  |  | < 0.001 |
| Upper | 260 (14.3) | 425 (25.8) |  |
| Middle | 216 (11.9) | 157 (9.6) |  |
| Lower | 1072 (59.1) | 789 (47.9) |  |
| Two-thirds or more | 266 (14.7) | 275 (16.7) |  |
| Tumor size |  |  | < 0.001 |
| ≤5cm | 933 (51.4) | 697 (42.3) |  |
| >5cm | 881 (48.6) | 949 (57.7) |  |
| Borrmann type |  |  | 0.111 |
| I+II | 1128 (62.2) | 980 (59.5) |  |
| III+IV | 686 (37.8) | 666 (40.5) |  |
| Histologic type |  |  | < 0.001 |
| G1+G2 | 362 (20.0) | 491 (29.8) |  |
| G3+G4 | 1452 (80.0) | 1155 (70.2) |  |
| T stage |  |  | < 0.001 |
| T1-2 | 693 (38.2) | 507 (30.8) |  |
| T3-4 | 1121 (61.8) | 1139 (69.2) |  |
| N stage |  |  | 0.059 |
| N0-N1 | 884 (48.7) | 855 (51.9) |  |
| N2-N3 | 930 (51.3) | 791 (48.1) |  |
| pTNM stage |  |  | 0.579 |
| Stage I+II | 869 (47.9) | 773 (47.0) |  |
| Stage III | 945 (52.1) | 873 (53.0) |  |
| Perineural invasion |  |  | 0.185 |
| Absence | 1544 (85.1) | 1374 (83.5) |  |
| presence | 270 (14.9) | 272 (16.5) |  |
| Lymphovascular invasion |  |  | 0.381 |
| Absence | 1490 (82.1) | 1333 (81.0) |  |
| presence | 324 (17.9) | 313 (19.0) |  |
|  | | | |
